# Letter from Samuel Cole, M.D., Heidelberg

**Published:** 1868-05-01

**Authors:** Samuel Cole

**Affiliations:** Heidelberg


### *FOREIGN CORRESPONDENCE.*

*To the Editor of the Chicago Medical Journal.*

HEIDELBERG, March 4, 1868.

DEAR SIR:—My attention being called to ovariotomy, by your editorial of January 15th, I resolved to collect the details of a case which had been treated in the hospital of this city, and report them to you. With this object in view, I waited upon Dr. N. Friederich, professor of general and special pathology and clinical medicine in the University of Heidelberg, and requested permission to make use of the hospital records. This the professor kindly granted to me, and after a similar interview with Dr. C. Heine, acting professor of surgery *pro tem.*, I am enabled to furnish you the following report:

Magdalen Maas, *æt.* 24, farmer's wife, of Seckenheim, was admitted to the hospital Dec. 16th, 1867. According to her own statement, she had always been healthy. In her nineteenth year, her menses made their appearance, and continued to recur regularly. At the time of her marriage (last January) she was not cognizant of any undue enlargement of the abdomen. Some eight to fourteen days after, she became conscious of pregnancy, which progressed normally. Four months before her confinement she experienced pain in the left leg. This continued four weeks, and then disappeared. An extraordinary size of the abdomen was noticed by the patient, who thought herself pregnant with twins. The delivery, on the 4th of October (ten weeks before her admission to the hospital), and subsequent convalescence, were perfectly normal. The size of the abdomen was reduced considerably, though not completely. It remained distended, and attained its present dimensions four weeks ago. Lochia normal. Menses did not re-appear. Appetite good, bowels regular.

Dec. 26th.—Patient complains of pain in the lumbar regions.

Dec. 28th.—Pain in the back has discontinued.

Dec. 29th.—A thorough physical examination yields the following *status præsens:* Abdomen distended, as if in the last month of gestation; the enlargement differing, however, from that of pregnancy, in being more in a transverse than in a longitudinal direction. Distinct fluctuation from every side, as if caused by ripples of large size, is every where present. No solid tumor and no fœtal parts can be felt, and no fœtal tones heard. On vaginal examination, the uterus is found in the median line, and measuring two and a half inches. The portio vaginalis is consistent, and bears marks of laceration. On percussion, flatness over the entire enlargement. Hypochondrii tympanitic. Along the left side of the abdomen tympanitic sound as low as the crest of the ilium, and remaining even when the patient lies on the left side. On the right, dullness on percussion. No boundary can be found as the flat percussion sound of the liver passes into that of the tumor. The lower edge of the liver can not be determined. Superiorly tympanitic sound. The line of separation of tympanitic and flat percussion sounds presents a curve whose convexity looks upward. No variation in the boundary line of flatness or tympanites on change of position. Urine has specific gravity of 1018. No albumen. Urates often present. The respiratory and circulatory organs normal. Diaphragm (right and left), in the fifth intercostal space.

Diagnosis—Unilocular ovarian cyst.

Jan. 14, 1868.—Temperature, which was carefully noted twice a day from the time of admission, averages 37.45 Cels., having at no time exceeded 39.00, nor been less than 36.20. The pulse ranged between 72 and 104, and averaged 87. The patient was transferred to the surgical wards to prepare for the operation of ovariotomy.

Jan. 16.—After the patient had been brought under the influence of chloroform, Dr. Heine made an incision about three inches in length, between the umbilicus and symphysis, in the linea alba, through the abdominal walls. This exposed the cyst. Spencer Wells' trocar was plunged into it. The walls of the cyst gave way more than necessary, and the fluid rushed forth at the side of the trocar. This was withdrawn, sharp hooks were fixed in the margins of the wound in the cyst, and in this manner it was prevented from retracting into the abdominal cavity as the fluid escaped. After the greater portion of the contents of the cyst had been evacuated, it was slowly drawn out. A few slight adhesions to the omentum on the right upper border were loosed with the finger without producing any hæmorrhage worthy of consideration. The omentum was then replaced, the pedicle of the cyst drawn out and fixed in Spencer Wells' clamp, the cyst amputated, and the stump cauterized with *ferrum candens.* The fluid which had escaped into the peritoneal cavity was removed by sponging, and the wound in the abdomen, which had contracted to about two inches, was closed by five simple sutures, the middle one including the peritoneum. Temp. 38.6 Cels. Pulse 108. Fifteen drops of Sydenham's *laudanum* administered. *Laudanum* to be repeated in the evening.

Jan. 17, A.M. Temp. 38.0; pulse 100. Patient slept during the night. Slight perspiration.

P.M., temp. 40.0: pulse 120. Urine drawn through catheter. Cataplasm over the abdomen.

Jan. 18, A.M., temp. 38.6; pulse 108. But little sleep. Perspiration more profuse. Slight eructation and occasional though transient feeling of weakness.

P.M., temp. 40.0; pulse 120. Umbilical region somewhat distended. Pulse small. *Tr. opii* gtt. x. administered. Sutures removed, and replaced by large strips of English adhesive plaster.

Jan 19, A.M., temp. 37.8; pulse 128. But little sleep. Abdomen tympanitic and moderately distended. Gurgling râles. Subjective symptoms wanting.

P.M., temp. 38.2; pulse 112. The edges of the wound looking well. Plaster renewed. In the evening a small quantity of thin fæces evacuated. The abdomen becomes remarkably less tense. Cataplasm repeated. *Tr. opii* gtt. xv. administered.

Jan. 20, A.M., temp. 37.8 ; pulse 100. Slept several hours last night, and feels tolerably well. Eructations almost completely gone. Thirst much less. Abdomen but slightly swollen in the region of the cæcum.

P.M., temp. 38.2 ; pulse 106. Patient removed to another bed, and laid upon an air pillow, as an extended erythema has made its appearance on the nates and over the sacrum.

From this time forward to the 26th, the temperature and pulse were normal, and there was one stool a day. On the 25th, the clamp, which hung only to a few necrotic shreds, was removed. On the right side of the fungiform pedicle, protruding from the wound, and at the base of the latter, is situated a funnel-shaped indentation, which was dressed with *chamomil. infus.* Pedicle itself touched with nitrate of silver. Patient coughs, and with much difficulty expectorates a little mucus. *Infus. Ipecacuan.* was prescribed.

Jan. 26. Vomited several times, after meals and during severe paroxysms of coughing. In the evening temp. 40.0 Cels. Pulse 112.

Jan. 27, A.M., temp. 37.8. Sputa yellow, viscid, pain in the chest. P.M., temp. 39.6.

Jan. 28, A.M., temp. 38.6. The examination of the thorax was made. On percussion : Dullness in a space of four fingers' breadth on the left side, posteriorly and inferiorly. On auscultation : Bronchial breathing, mucous râles. On palpitation : Diminished pectoral fremitus. No pain. Viscid, slimy sputa ; patient feels weak. Lips, pale, livid. No dyspnœa nor chilliness. Abdomen soft. Wound looking well, pedicle moderately suppurating, shrunken, and retracted to the level of the abdomen. Wine *decoct. quiniæ* prescribed.

P.M., temp. 40.2 ; pulse 124. Twice vomiting during the day of food which she had taken. Bitter taste in the mouth. Occasional eructation, frequent cough, sputa brick dust color. No dyspnœa. Cheeks red. Two watery stools in the course of the day. No albumen in the urine. *Decoct. quin.* discontinued.

Jan. 29, A.M., temp. 38.4 ; pulse 112. Patient complains of continual and severe irritation to cough. Four diarrhœal stools. Vomited once during the night. Sputa only slightly slimy. Feeling of weakness. On percussion, dullness on the left side, posteriorly and inferiorly, as yesterday. Bronchial breathing more marked. Abdomen slightly distended. Wound granulating nicely, Dressing as heretofore. Prescription of *Flor. benzoiac* 5 grs. every hour. *Tr. opii* 15 drops.

P.M., temp. 40.0. During the day vomited once; had one stool more. Feels easier, coughs less; abdomen still pliable.

Jan. 30, A.M., temp. 38.2; pulse 112. Dullness somewhat higher. Pleuritic friction sound. Increased weakness.

P.M., temp. 39.0; pulse 136. Somewhat more mucus expectorated. Dullness left, posteriorly and inferiorly clearing. Patient always vomits after meals.

Jan. 31, A.M., temp. 37.8; pulse 104. Patient had a very restless night. Eight thin evacuations resembling coffee grounds; restlessness; mouth dry. Expectoration less; no vomiting. Champagne prescribed, and continued from this time forward.

P.M., temp. 40.0; pulse 114. During the day no stool, no vomiting, although she has frequently partaken of broth and egg. Dyspnœa less. Cough and expectoration diminished. Pulse small.

Feb. 1, A.M., temp. 38.0; pulse 116. Very restless during the night. One stool, and vomited once. Cough and expectoration almost gone. Sputa reddish. Weakness great, but not increased. Dullness on percussion on the left side from below upward to about one half the height of the lung, and bronchial breathing above. On the right side rhonchi sonori, percussion normal. Vesicular respiration at the apex. *Amylon enema* prescribed.

P.M., temp. 39.0; pulse 120. Three diarrhœal stools. But little sputa. Physical condition the same. Beginning decubitus. Wound dressed with *Ung. præcip.*

Feb. 2, A.M., temp. 38.6. During the night several thin stools, vomited several times, and slept but little. Examination of the thorax reveals nothing new. Right side, rhonchi. Dyspnœa, cough and expectoration as heretofore. Wound not granulating so well. Dressed as heretofore.

P.M., temp. 39.0. During the day a number of thin stools. Vomited a fluid of green color. Tongue dry. Decubitus somewhat more marked.

Feb. 3, A.M., temp. 38,0. P.M., temp. 38.6; pulse 132. Had four stools during the night, and vomited twice. *Pot. Riveri* ℥ v. with *Aqua lauro-cerasi* ʒ i. prescribed in tablespoonful doses, and Liebig's essence of beef, tablespoonful every hour. Vomited twice (once bile) during the day. The left parotid somewhat tender and swollen. *Pot. Riveri*, tablespoonful.

Feb. 4, A.M., temp. 37.8; pulse 112. During the night vomited once; three stools.

P.M., temp. 37.6; pulse 96. During the day vomited once. One stool. Pulse somewhat fuller. Liebig's essence of beef given as food. *Pot. Riveri* and champagne continued.

Feb. 5, A.M., temp. 38.6. Increasing collapse, general and local.

P.M., temp. 38.4. During the day, neither stool nor vomiting. Right iliac region somewhat tender.

Feb. 6, A.M., temp. 38. During the night, neither stool nor vomiting. Collapse increases. Afternoon, temp. 38.5. Weakness very great; pulse small. Night, temp. 34. Respiration short and frequent. Sensorium involved. Death, 12 P.M.

Anatomical Diagnosis.—Peritonitis diffusa, encapsuled collections of pus in the vesico and recto-vaginal culs-de-sac (Douglas's). Lobular accumulations in the left lung, fibrinous pneumonia of the right. Acute intestinal catarrh and parenchymatous degeneration of the abdominal glands.

This report, taken from the hospital records, calls for a few general deductions, which, with your permission, I shall attempt to make. The diagnosis of the case was correct, the patient in good condition, and the prognosis favorable. The operation was performed in a masterly manner, without having encountered any peculiar or unexpected difficulty. The prognosis might have been better had not a small quantity of the fluid contents of the cyst escaped into the peritoneal cavity. This, however, was removed, and if ever there was a case in which the hope of a speedy recovery might reasonably be entertained, it was this. Yet the patient succumbed in three weeks.

In the editorial to which I have already referred, you advance that, the greater the departure of the peritoneum from its normal condition, the less the danger of interfering with it. This may be true, but we must bear in mind that the greater the change in the peritoneum, the more extensive also will be the adhesions. You do not consider adhesions as unfavorable, but I must regard them as, at least, very unpleasant complications, for they require a longer abdominal wound, serve to protract the operation, and thus increase the shock to the general system. What on the one hand would be gained by lessening the chances of peritonitis, would on the other, be more than compensated for by the more frequent and more severe shock of the operation.*

In commenting on the case before us, it is not my purpose to discuss whether the conditions would have been more favorable had there been more adhesions, or had there been a greater degeneration of the peritoneal membrane, nor to inquire into the immediate cause of the fatal termination, if produced by peritoneal inflammation, or by consecutive pneumonia; it is enough to know that the patient died, and that the fatal issue is a consequence of the operation.

Dr. B. Stilling, of Cassel, in a report on ovariotomy (*Deutsche Klinik* of Jan. 18th, 1868), gives the particulars of a case of multilocular ovarian cyst of ten years' standing, which had been tapped seven times. The patient was well nourished. Ovariotomy was performed, and the patient died in three days. He also reports a case (Feb. 15th), of multilocular cyst (ovarian) of two years' standing, emaciation and

* The opinion of the JOURNAL is that the shock is also less when there is great departure from the normal condition.—ED.

failure of the vital powers keeping pace with the growth of the tumor. Ovariotomy was performed, and the recovery complete in eighteen days.

In the former of these two cases, the general health was good, and no operation was called for as a matter of necessity. In the latter, where the vital powers were sinking, an operation was decidedly indicated.

The results of the operations in these cases, accidentally favor the position I take in the question of what are the indications for operation. In my opinion, the sinking of the vital powers, increasing emaciation, etc., are the true indications; not that they afford any better prospect of success, nor that they facilitate the operation; on the contrary, they make recovery more doubtful, and the operation more difficult. But, in the mean time, the patient is permitted to live (with now and then a paracentesis), in comparative comfort, for an interval of seven, ten, or perhaps twelve years. Is the percentage of recovery *so* great as to justify us in performing an ovariotomy on a person who bids fair to live a number of years without this most dangerous proceeding? The difficulties of the operation are increased by delay, but are we to imperil the lives of those who confide in us, merely because we are too timid to undertake an operation more difficult of execution? The dangers also are increased — those from shock, in particular. Possibly, those from inflammatory process in the peritoneal sac are lessened. Be that as it may, the records do not demonstrate that the advantages of early operations over those deferred to a later period are *marked* enough to encourage us in performing them.

Many cases, in which the ovarian cyst, after paracentesis, requires only a short space of time to refill, do not admit of delay for a number of years. The strength of the patient rapidly declines, the system not being able to sustain the repeated and exhausting efforts it is called upon to make. In these cases we should convince ourselves of the futility of paracentesis, and *then* proceed to operate before the vital powers are reduced to their lowest ebb. The indication remains the same, the failure of the general health, sooner or later, marking the time when it is our duty to operate.

In this synopsis you will perceive that the patient was well nourished, the cyst unilocular, and containing a serous fluid. It was not tapped in order to avoid forming adhesions. If it had been, it probably would have refilled in a very short time, judging from the brief period it required for arriving at its former dimensions. Still, the possibility remains that the fluid might have been slow in accumulating, and the tumor have been tapped for a number of years. The dangers of the final, radical operation would have been increased, without doubt, but not so materially but that it could have been undertaken with a fair prospect of success.

Yours truly, SAMUEL COLE, M.D.

---

